# How well do obesity indices predict undiagnosed hypertension in the Persian cohort (Shahedieh) adults community population of all ages?

**DOI:** 10.1002/hsr2.1897

**Published:** 2024-02-23

**Authors:** Sara Jambarsang, Moslem Taheri Soodejani, Robert Tate, Reyhane Sefidkar

**Affiliations:** ^1^ Center for Healthcare Data Modeling, Departments of Biostatistics and Epidemiology Shahid Sadoughi University of Medical Sciences Yazd Iran; ^2^ Centre on Aging University of Manitoba Winnipeg Canada; ^3^ Department of Community Health Sciences, Max Rady College of Medicine University of Manitoba Winnipeg Canada

**Keywords:** anthropometry, hypertension, obesity indices, primary prevention, ROC curve

## Abstract

**Background and Aims:**

Hypertension is the leading preventable risk factor for cardiovascular disease, chronic kidney disease and cognitive impairment, and mortality and disability worldwide. Since prevention, early detection, and treatment of blood pressure improve public health, the aim of present study was to determine the best obesity indices and estimate the optimal cut‐off point for each one to predict the risk of elevated/stage 1 and undiagnosed hypertension in the population of center of Iran based on American ACC/AHA 2020 guidelines.

**Methods:**

This cross‐sectional study was performed on 9715 people who enrolled in 2018 in Persian Adult Cohort in Shahedieh area of Yazd, Iran in 2018. The anthropometric indices including body mass index (BMI) and waist circumference (WC), wrist circumference, hip circumference, waist‐to‐hip ratio, and waist‐to height ratio of individuals, were extracted. The receiver operating characteristic curve was utilized to determine the optimum cut‐off point of each anthropometric index to predict hypertension stages and compare their predictive power by age‐sex categories. Statistical analysis was done using SPSS version 23.0.

**Results:**

The results showed that BMI has the best predictive power to recognize the risk of elevated/stage 1 hypertension for female (area under the curve [AUC] = 0.72 and optimal cut‐off = 30.10 kg/m^2^) and WC for male (AUC = 0.66 and optimal cut‐off = 93.5 cm) in 35−45 age group. BMI had the best predictive power for the risk of undiagnosed hypertension for 35−45 years old male (AUC = 0.73 and optimal cut‐off = 28.90 kg/m^2^) and female (AUC = 0.75 and optimal cut‐off = 5.10 kg/m^2^), and hip circumference revealed similar predictive power for female as well (AUC = 0.75 and optimal cut‐off = 112 cm).

**Conclusion:**

Based on our findings, BMI and WC, which are simple, inexpensive, and noninvasive means, are the best markers to predict the risk of elevated/stage 1 and undiagnosed hypertension in young Iranians. It shows that the approach of reducing hypertension prevalence through primary prevention, early detection, and enhancing its treatment is achievable.

## 1 INTRODUCTION

Hypertension is a global public health problem which is considered as one of the most common noncommunicable health disorders.^1^, ^2^ Over the last decades, the prevalence of hypertension has been increased around the world due to the rapid socioeconomic and lifestyle changes. According to the recent estimates, about one‐third of the adult world population (1.39 billion) have hypertension which is more prevalent in low‐ and middle‐income countries.^3^, ^4^ Hypertension is one of the major causes of morbidity and mortality in the world.^5^ Based on World Health Organization (WHO) reports, hypertension is responsible for one out of eight mortalities worldwide.^4^ Hypertension may cause other significant complications such as stroke, coronary artery disease, progression of chronic kidney disease, heart failure in patients as well.^1^ It is estimated that more than one and a half billion people worldwide will suffer from high blood pressure by 2025.^6^

Globally, noncommunicable diseases are recognized as the main cause of death. These are responsible for about 70% of all deaths; of whom one‐third are 69 years or younger. Some of those diseases start without symptoms, such as high blood pressure.^7^ Fortunately, high blood pressure can be diagnosed in the community and in primary care facilities. There are low cost but effective drugs to treat patients with hypertension and decline the risk of further complications associated with hypertension.^8^

Given that regular screening is required for early detection of hypertension, it can remain unknown and therefore remain untreated in some occasions. According to the 2020 AHA hypertension guideline, elevated status (120−129 systolic and <80 mmHg diastolic) and stage 1 hypertension (130−139 systolic or 80−89 mmHg diastolic) can be improved without the need for immediate medical treatment and simply by changing lifestyle,^9^ such as weight control, smoking cessation, reducing consumption alcohol and salt, also do proper physical activity.^10^ In case of stage 2 of hypertension (systolic pressure ≥140 mmHg or diastolic pressure ≥90 mmHg), people need medical intervention. However, if the hypertensive condition is not diagnosed by a physician, the person may stay with this condition for a long time. Undiagnosed hypertension is a crucial risk factor for kidney and cardiovascular disease.^2^ When people are unaware that they have high blood pressure and accidentally find out about it on an examination, it is called undiagnosed hypertension.^9^

The problem of obesity and its comorbidities continues to rise around the world and is becoming an epidemic.^11^ Reports and estimates from health institutions shows that obesity, with prevalence of about 1.9 billion adults in 2016, could reach about 18% in men and 21% in women by 2025.^12^ There is ample evidence to support a link between obesity and hypertension.^13^, ^14^ Many studies have explain the mechanism of this relationship.^15^ In clinical practice, anthropometric indices such as body mass index (BMI) and upper‐body adiposity (waist circumference [WC], waist‐to‐hip ratio [WHR], and waist‐to height ratio [WHtR]) still have an important role to measure total body fat and its distributions.^16^ Based on the literature, the association between these measurements and cardiovascular disease risk factors such as hypertension in all ethnic and age‐sex groups, because of physiological factors, is not ignorable.^16^, ^17^

Regarding the impact of high blood pressure and its sequels on health outcomes, it seems that by regular screening and early detection of undiagnosed hypertension, public health could improve and the cost of healthcare could reduce.^2^ Therefore, the primary objective of this study is to estimate the prevalence of undiagnosed hypertension and elevated/stage 1 hypertension in Iranian population. We also recognize the best anthropometric indices and detect the optimal cut‐off point for each of these indices by age‐sex grouping to predict hypertension status using receiver operating characteristic (ROC) curve method in the population of a central city in Iran.

## 2 METHODS AND MATERIALS

### 2.1 Study design

In this cross‐sectional study, we used the data from recruitment phase of Yazd (Shahedieh) cohort study in center of Iran in 2018. The Shahedieh study is a part of a prospective epidemiological research study in Iran (PERSIAN cohort).^18^ Shahedieh cohort study recruited about 10,000 adults between the ages of 35 and 70 (both men and women) living in two municipal areas of Yazd city (Zarch and Shahedieh), Yazd province, Iran. The study protocol of the PERSIAN cohort is provided in more detail, elsewhere.^18^, ^19^ All the participants that met the inclusion criteria were provided oral and written informed consent. This study was in accordance with the Declaration of Helsinki and was registered with the Center for Healthcare Data Modeling and approved by the Ethics Committee of Shahid Sadoughi Yazd University of Medical Sciences (IR.SSU.SPH.REC.1401.089).

### 2.2 Sample size and participants

**Figure 1:**
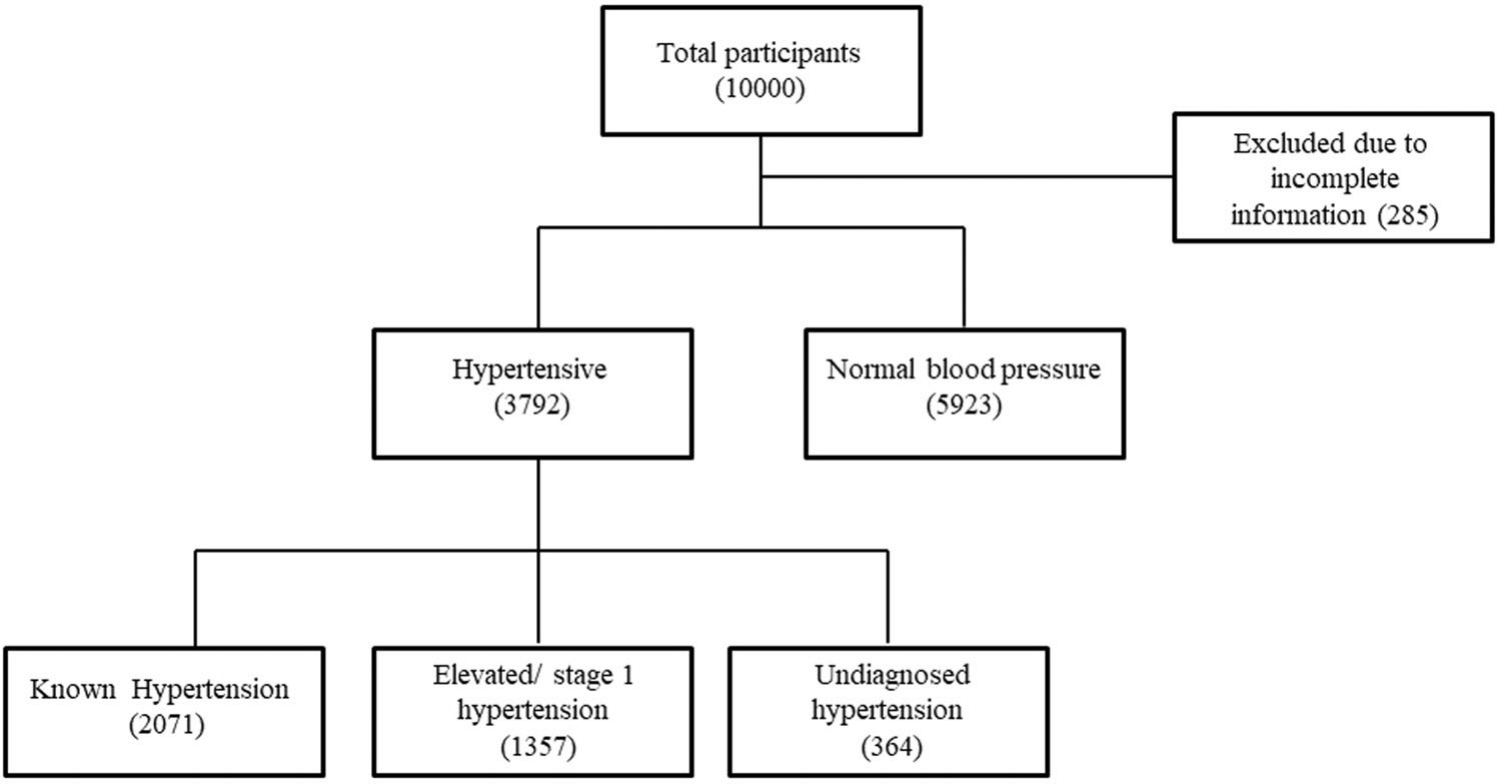
Flowchart of Shahedieh cohort participants.

Data of 9968 adults were included. Subjects whose blood pressure data were incomplete, 253 subjects, are excluded from the present study. As a result, the data of 9715 people were analyzed (Figure 1).

### 2.3 Anthropometric measurement

Anthropometric parameters (weight, height, hip circumference, WC, and wrist circumference) were measured by a trained researcher. Weight was measured while the subjects were with minimum clothing and without shoes using a digital scale (SECA; model 755). Hip circumference and WC were measured while participants wear minimum cloth. Height of subjects was measured by a tape measure attached to the wall without any bumps with a precision of 0.5 cm.^18^ The BMI, WHR, and WHtR was calculated using SPSS version 23.0 for windows (SPSS Inc.).

### 2.4 Blood pressure definition

Participant systolic (SBP) and diastolic (DBP) blood pressure was measured in a seated position, after at least a 10 min rest period. Two separate measurements were recorded from both right and left arm using a mercury sphygmomanometer. The final blood pressure was obtained by averaging the second right and left hand blood pressure measurements. People with self‐reported high blood pressure or were taking antihypertensive drugs, were defined as “known hypertension” category. Participants with SBP ≥ 140 mmHg or DBP ≥ 90 mmHg defined as hypertensive.^9^ Participants who were first diagnosed with hypertension by the study, were categorized as “undiagnosed hypertension” status. Based on new definition of blood pressure categories, elevated category includes subjects with 120−129 SBP and <80 mmHg DBP, also the stage 1 hypertension includes subjects with 130−139 systolic or 80−89 mmHg diastolic.^9^ In this study, the last two categories were considered together as a group, “elevated/stage 1 hypertension,” because both groups need to change their lifestyle to prevent progression to hypertension.^9^ Other subjects, with SBP < 120 and DBP < 80 mmHg, were assumed to have “normal blood pressure” based on American ACC/AHA 2020 guidelines.^9^

### 2.5 Statistical analysis

Data were analyzed using the statistical software SPSS version 23.0 for windows (SPSS Inc.) and “OptimalCutpoints” package of R software version 4.2.0. Quantitative data were summarized using mean (standard deviation [SD]). Qualitative data have been described by frequency (%). The optimal cut‐off values of anthropometric indices and age to predict hypertension statuses were determined using ROC.^20^ The area under the ROC curve (AUC) was also measured to assess and compare the predictive power of the anthropometric indices and age. Optimal cut‐off values of indices were determined by calculating the sensitivity and specificity of the anthropometric measurements at various cut‐off points.^21^ Data analyzes were conducted by sex and age group ignoring known hypertension category.

## 3 RESULTS

The study population included 4865 males and 4850 females, of whom 1019 males and 1046 females had the history of hypertension. The means and SDs of age and anthropometric markers by hypertension status are presented in Table 1. According to the status of hypertension, 21.3% of adult ≥35 years old were hypertensive and 3.7% of subjects have undiagnosed hypertension. Undiagnosed hypertension or elevated/stage 1 hypertension is more prevalent in older and male participants. A higher BMI in this community is evident in undiagnosed hypertension category and elevated/stage 1 hypertension status with mean (SD), 29.7 (4.9) and 31 (5.4), respectively. As expected, subjects in the undiagnosed or elevated/stage 1 hypertension category had larger hip circumferences, weight, WC, and wrist circumference compared with known hypertensive or normal blood pressure category.

**Table 1:** Anthropometric characteristics of participants in Shahedieh cohort by hypertension status.

	Known hypertension (*N* = 2071)	Normal blood pressure (*N* = 5923)	Elevated/stage 1 hypertension (*N* = 1357)	Undiagnosed hypertension (*N* = 364)
Age (years)	49.4 (9.84)	46.70 (9.05)	54.10 (9.18)	52.40 (9.07)
Male (%)	1019 (49.30)	2858 (48.30)	774 (57.00)	214 (58.80)
Weight (kg)	75.61 (13.42)	74.54 (13.45)	79.16 (14.44)	83.28 (15.84)
Height (cm)	162.92 (9.57)	163.43 (9.49)	163.30 (10.04)	163.81 (10.05)
Hip circumference (cm)	102.96 (8.96)	102.49 (9.06)	104.38 (9.84)	106.58 (10.49)
WC (cm)	96.41 (11.09)	94.65 (11.35)	100.27 (11.26)	103.15 (11.82)
Wrist circumference (cm)	17.46 (1.49)	17.23 (1.45)	17.89 (1.48)	18.18 (1.63)
BMI	28.54 (4.79)	27.95 (4.72)	29.71 (4.92)	31.05 (5.39)
WHR	0.94 (0.07)	0.92 (0.07)	0.96 (0.06)	0.97 (0.06)
WHtR	0.59 (0.07)	0.58 (0.07)	0.62 (0.08)	0.63 (0.08)

Abbreviations: BMI, body mass index; WC, waist circumference; WHR, waist‐to‐hip ratio; WHtR, waist‐to height ratio.

**Figure 2:**
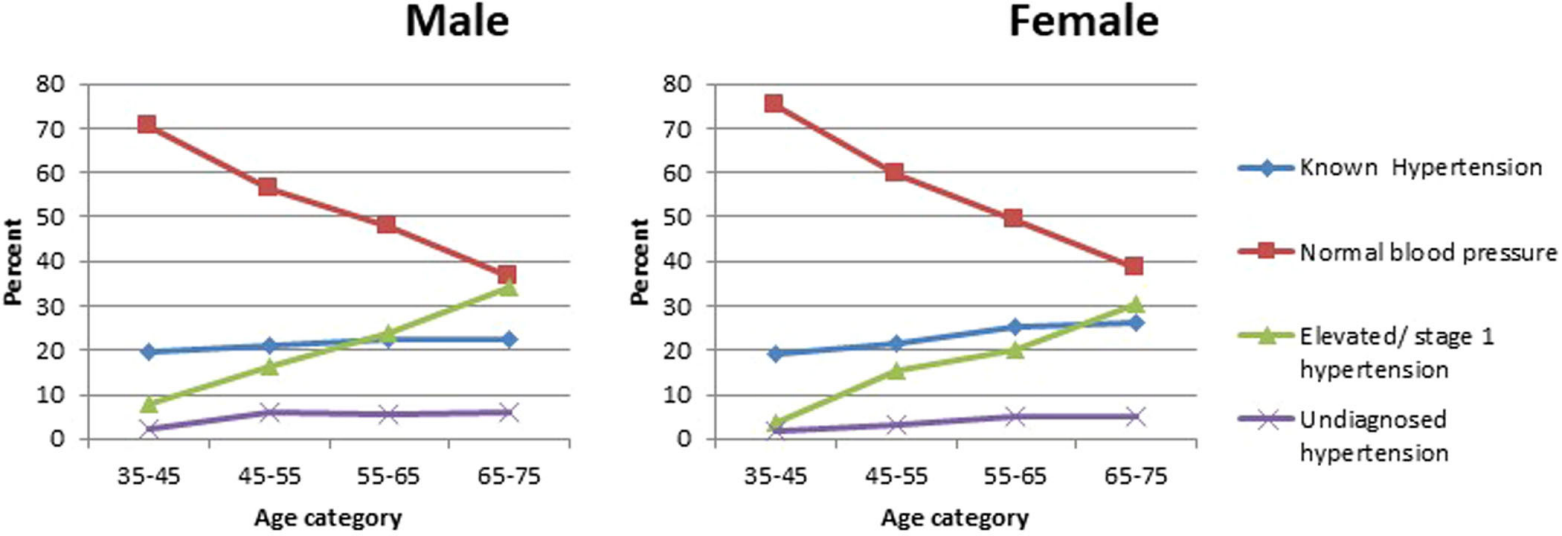
Prevalence of hypertension status for different age‐sex groups according to American ACC/AHA guidelines.

In female, elevated/stage 1 hypertension prevalence increases until 45−55 age group. This prevalence gets close at 55−65 age category to known status prevalence and they seem so similar at the last group. This prevalence has upward trend until 55−65 age category in male. It is worth to mention that the prevalence of this status is higher than known hypertension status in this age range. Percent of hypertension stages by sex and age groups is shown in Figure 1. Percent of undiagnosed hypertension is less than other statuses in all ages in both gender. Although this percentage is steady over ages in female, it has increased to some extent in male at 45−55 years. However the prevalence of undiagnosed hypertension in both sexes is low in 65−75 years age group, it is lower in female in the mentioned age group (Figure 2).

BMI (cut‐off point = 30.10 kg/m^2^, AUC = 0.72; 95% CI: [0.67−0.77]) and WHtR (cut‐off point = 0.61, AUC = 0.70; 95% CI: [0.66−0.76]) had the most power to predict elevated/stage 1 hypertension for 35−45 year females. While in this age category, WC has the highest AUC in males (cut‐off point = 93.50 cm, AUC = 0.66; 95% CI: [0.61−0.70]).

According to AUC which was used to estimate and compare the predictive power of anthropometric indices, the WHtR (cut‐off point = 0.66, AUC = 0.59; 95% CI: [0.56−0.64]) and WC (cut‐off point = 0.95, AUC = 0.59; 95% CI: [0.55−0.63]) are the best predictor for the risk of elevated/stage 1 hypertension among other anthropometric indices in 45−55 year females. Furthermore, in 45−55 year males, BMI (cut‐off point = 25 kg/m^2^, AUC = 0.64; 95% CI: [0.60−0.68[) and weight (cut‐off point = 81 kg, AUC = 0.64; 95% CI: [0.59−0.67]) had similar predictive for this risk.

Despite that the predictive power of all anthropometric indices decrease in 55−65 category, BMI, WC, weight, hip circumference, wrist circumference in males, and WHtR in female still have acceptable power, however, all the markers lose their effectiveness for prediction of elevated/stage 1 hypertension in both sexes (Table 2).

**Table 2:** The areas under ROC curve (AUC), optimal cut‐off values, sensitivities and specificities of age, and anthropometric markers associated with elevated/stage 1 hypertension by age group.

		Age group							
		35−45 (*N* = 4104)		45−55 (*N* = 2980)		55−65 (*N* = 2049)		65−75 (*N* = 582)	
Marker	Gender	Cut‐off point	AUC (95% CI)	Cut‐off point	AUC (95% CI)	Cut‐off point	AUC (95% CI)	Cut‐off point	AUC (95% CI)
Weight (kg)	Female	71.00	0.69 (0.63−0.75)	86.50	0.56 (0.52−0.60)	67.50	0.52 (0.48−0.57)	66.00	0.54 (0.46−0.62)
	Male	79.00	0.65 (0.60−0.69)	81.00	0.64 (0.59−0.67)	73.00	0.57 (0.53−0.61)	78.00	0.53 (0.46−0.59)
Hip circumference (cm)	Female	110.20	0.66 (0.60−0.72)	112.00	0.55 (0.51−0.59)	113.00	0.53 (0.48−0.57)	114.00	0.54 (0.46−0.62)
	Male	102.30	0.64 (0.59−0.68)	101.20	0.62 (0.58−0.66)	94.50	0.55 (0.51−0.59)	105.40	0.52 (0.45−0.59)
WC (cm)	Female	96.50	0.69 (0.64−0.75)	95.00	0.59 (0.55−0.63)	98.00	0.54 (0.49−0.58)	100.00	0.57 (0.49−0.65)
	Male	93.50	0.66 (0.61−0.70)	97.70	0.63 (0.59−0.67)	98.60	0.55 (0.51−0.59)	114.00	0.51 (0.44−0.58)
Wrist circumference (cm)	Female	16.50	0.67 (0.61−0.73)	15.60	0.57 (0.53−0.61)	16.80	0.53 (0.48−0.57)	16.60	0.56 (0.48−0.63)
	Male	18.00	0.62 (0.57−0.67)	19.70	0.59 (0.55−0.63)	18.40	0.56 (0.52−0.59)	17.50	0.57 (0.50−0.63)
BMI	Female	30.10	0.72 (0.67−0.77)	28.60	0.58 (0.54−0.62)	29.70	0.54 (0.49−0.58)	32.40	0.53 (0.45−0.61)
	Male	29.30	0.64 (0.59−0.69)	25.00	0.64 (0.60−0.68)	24.10	0.55 (0.51−0.59)	24.80	0.51 (0.44−0.58)
WHR	Female	0.87	0.64 (0.58−0.69)	0.94	0.57 (0.53−0.61)	0.96	0.52 (0.48−0.57)	0.88	0.51 (0.44−0.59)
	Male	0.93	0.64 (0.59−0.69)	0.92	0.61 (0.57−0.64)	0.92	0.53 (0.49−0.56)	0.97	0.49 (0.43−0.56)
WHtR	Female	0.61	0.70 (0.65−0.76)	0.66	0.59 (0.56−0.64)	0.65	0.55 (0.51−0.59)	0.77	0.56 (0.48−0.64)
	Male	0.55	0.65 (0.60−0.69)	0.54	0.63 (0.59−0.66)	0.49	0.54 (0.49−0.58)	0.56	0.49 (0.43−0.56)

Abbreviations: BMI, body mass index; ROC, receiver operating characteristic; WC, waist circumference; WHR, waist‐to‐hip ratio; WHtR, waist‐to height ratio.

Similar to elevated/stage 1 hypertension status, the best predictions were observed in 35−45 age range in undiagnosed hypertension status. BMI (cut‐off point = 35.10 kg/m^2^, AUC = 0.75; 95% CI: [0.68−0.83]) and weight (cut‐off point = 80 kg, AUC = 0.75; 95% CI: [0.68−0.83]) of females like the males (cut‐off point = 28.90, AUC = 0.73; 95% CI: [0.66−0.81] for BMI and cut‐off point = 84 kg, AUC = 0.72; 95% CI: [0.64−0.81] for weight), have the highest. Hip circumference (cut‐off point = 112 cm, AUC = 0.75; 95% CI: [0.68−0.83]) has effective power for prediction in female of this category, as well. WC in 45−55 (cut‐off point = 91 cm, AUC = 0.67; 95% CI: [0.62−0.72]) and 55−65 (cut‐off point = 99 cm, AUC = 0.66; 95% CI: [0.59−0.72]) years old males was the best index to predict undiagnosed hypertension status. For 45−55 years old females, hip circumference (cut‐off point = 107.40 cm, AUC: 0.64; 95% CI: [0.56−0.71]) and for 55−65 years old WHtR (cut‐off point = 0.66, AUC = 0.71; 95% CI: [0.63−0.79]) are the best. Except WHtR for females (cut‐off point = 0.64, AUC = 0.64; 95% CI: [0.57−0.71]), none of the indices is recommended to predict undiagnosed hypertension status for oldest in both sexes (Table 3).

**Table 3:** The areas under ROC curve (AUC), optimal cut‐off values, sensitivities and specificities of age, and anthropometric markers associated with undiagnosed hypertension by age group.

		Age group							
		35−45 (*N* = 4104)		45−55 (*N* = 2980)		55−65 (*N* = 2049)		65−75 (*N* = 582)	
Marker	Gender	Cut‐off point	AUC	Cut‐off point	AUC	Cut‐off point	AUC	Cut‐off point	AUC
Weight (kg)	Female	80.00	0.75 (0.68−0.83)	79.00	0.62 (0.54−0.70)	78.00	0.64 (0.56−0.73)	72.00	0.53 (0.36−0.70)
	Male	84.00	0.72 (0.64−0.81)	78.00	0.66 (0.60−0.71)	75.50	0.64 (0.57−0.71)	74.00	0.51 (0.39−0.63)
Hip circumference (cm)	Female	112.00	0.75 (0.68−0.83)	107.40	0.64 (0.56−0.71)	107.00	0.61 (0.53−0.69)	105.00	0.49 (0.33−0.65)
	Male	104.00	0.71 (0.62−0.80)	99.80	0.64 (0.58−0.69)	99.00	0.62 (0.55−0.69)	92.00	0.49 (0.36−0.61)
WC (cm)	Female	99.00	0.72 (0.64−0.80)	103.80	0.64 (0.56−0.71)	104.00	0.67 (0.59−0.75)	102.00	0.55 (0.41−0.70)
	Male	92.60	0.69 (0.62−0.77)	91.00	0.67 (0.62−0.72)	99.00	0.66 (0.59−0.72)	99.00	0.55 (0.42−0.67)
Wrist circumference (cm)	Female	17.60	0.65 (0.56−0.74)	18.40	0.56 (0.48−0.65)	17.00	0.66 (0.58−0.74)	17.50	0.57 (0.37−0.78)
	Male	18.40	0.71 (0.62−0.79)	18.10	0.61 (0.55−0.66)	18.30	0.65 (0.57−0.72)	18.60	0.49 (0.34−0.64)
BMI	Female	35.10	0.75 (0.68−0.83)	31.20	0.63 (0.55−0.71)	32.80	0.68 (0.59−0.75)	30.20	0.59 (0.42−0.75)
	Male	28.90	0.73 (0.66−0.81)	28.60	0.66 (0.60−0.71)	26.30	0.64 (0.58−0.71)	22.90	0.51 (0.38−0.64)
WHR	Female	0.88	0.56 (0.46−0.65)	0.97	0.56 (0.48−0.64)	0.98	0.60 (0.52−0.69)	1.02	0.59 (0.43−0.76)
	Male	0.90	0.61 (0.53−0.69)	0.92	0.65 (0.59−0.70)	0.96	0.63 (0.56−0.69)	0.93	0.57 (0.44−0.69)
WHtR	Female	0.66	0.71 (0.63−0.79)	0.62	0.54 (0.40−0.68)	0.66	0.71 (0.63−0.79)	0.64	0.64 (0.57−0.71)
	Male	0.53	0.69 (0.62−0.76)	0.55	0.66 (0.61−0.72)	0.57	0.64 (0.58−0.70)	0.62	0.54 (0.40−0.68)

Abbreviations: BMI, body mass index; ROC, receiver operating characteristic; WC, waist circumference; WHR, waist‐to‐hip ratio; WHtR, waist‐to height ratio.

## 4 DISCUSSION

To date, few studies have been conducted on undiagnosed hypertension divided by sex‐age categories, worldwide. The primary aim of this cross‐sectional study is assessing the prevalence of undiagnosed and elevated/stage 1 hypertension and its related obesity factors optimal cut‐off points to predict it among Iranian adults. This research revealed that the prevalence of known hypertension was 21% and the prevalence of undiagnosed hypertension was 3.7% which is so various in the literature. The undiagnosed prevalence of hypertension are reported from 10% to more than 50%.^22^, ^23^ About 29% of adults in the United States has been affected by hypertension. A report from National Health and Nutrition Examination Survey in 2007–2008 showed that 20% of adults are unaware of their diagnosis of hypertension.^24^ In a study in Saudi Arabia, the hypertension prevalence was reported to be 14%–41.8% and among hypertensives, 27.6%–61.1% were aware of their status.^25^ The results of a meta‐analysis in India showed that the overall prevalence of hypertension in India was 29.8%.^26^ Another study in India estimated the age‐adjusted prevalence of hypertension about 11.3% among persons aged 15–49.^27^ The prevalence of undiagnosed hypertension in a study in Southern Ethiopia was reported 12.3%.^28^ In another study which was conducted in United Kingdom, the untreated hypertension prevalence was estimated 5%.^29^ It is deduced that the observed prevalence of undiagnosed hypertension (nearly 4%) showed that the community is in a good condition in terms of blood pressure screening. Furthermore, studying the effectiveness of these markers and determining the optimal cut‐off points to predict hypertension levels in different sex‐age groups was considered.

Totally, findings showed higher risks of elevated/stage 1 and undiagnosed hypertension among males who are older in age than females, although this trend is increasing in females as well. This high prevalence of elevated/stage 1 and undiagnosed hypertension at older ages identified in the current assessment may be due to people's lack of knowledge to receive regular health check‐ups, coupled with dwelling in the rural area.^30^ Based on the results, it was observed that the prevalence of elevated/stage 1 hypertension in male is twice the female in 35–45 age category. According to the literature, risk of cardiovascular disease is higher in men than in age‐matched premenopausal women.^31^ Findings of another study which was conducted on urban population of Yazd, Iran indicated that prevalence of prehypertension was 42.50% among males and 33.70% among females. The results of this study showed that this prevalence approximately decreases by the increase in age (45.10% in 30–39 years to 24.40% in 60–69 years).^1^ In the other study in Turkey, the prevalence of prehypertension was reported 16.80% for males and 12.60% for females. Furthermore, this parameter was less in the elderly in both sexes (17.70% and 15.20% in 30–39 years males and females, respectively, and 8.50% and 8.20% in 70+ years in males and females).^32^

In our study, all anthropometric indices were known as useful screening tools for diagnosis of elevated/stage 1 and undiagnosed hypertension in under 55 years old participants, especially in 35–45 age group. It seems that the low predictive power of biomarkers in old age is more affected by aging and physiological factors than obesity indices.^17^

Based on various studies, it can be remarked that choosing the best index for predicting blood pressure depends on ethnicity and race, so that in a study which was conducted in India, BMI has been suggested as the best index^33^ and in another study in Iraq, WHtR was identified as the most predictive marker.^16^ A study in Turkey also detected BMI as the best predictor for hypertension.^34^ However, none of them considered the effect of age, our study showed that BMI is a suitable index to diagnosis of elevated/stage 1 and undiagnosed hypertension only in women aged 35–45. This index, which is closely related to the total amount of body fat and is used to define the criteria of overweight or obesity,^35^ is useful to predict undiagnosed hypertension in 35–45 years old males. WC was also the best predictor for elevated/stage 1 hypertension status in the mentioned category. WHtR has strong association with elevated/stage 1 hypertension status in 35–45 years old females. Several systematic reviews and primary studies also have recommended WHtR as a screening tool.^36^

Given that anthropometric indices depend on the climate, nutrition, and culture of each community, it is necessary to determine the appropriate index for hypertension screening in each thematic community. However, the present study showed similar indicators to previous studies. Although recognition of appropriate markers to detect elevated/stage 1 hypertension is so important, determination of appropriate cutting points, which is rarely addressed in the literature, is necessary too. Because people at this level can return to normal blood pressure level simply by changing their lifestyle, doing physical activity, and without the need for treatment, screening in this stage is important.^9^ Among the anthropometric indices studied in this study, WHR for men and WHtR for women are the best indicators to diagnose elevated/stage 1 hypertension stage.

As the optimal cut‐off of obesity indices was estimated divided by age‐sex groups, our results are not comparable to other studies. The optimal cut‐off values of WHtR in our study was 0.61 in 35–45 years old women, which was different from an study in Asia, India ignoring age.^33^ BMI is always considered as a well‐known index for the general public. In the present study, the calculated cut‐off point for this index was 35.1 kg/m^2^ in women and 28.901 kg/m^2^ in men to predict undiagnosed hypertension in 35–45 age group. The WHO, meanwhile, has set a cut‐off point of 30 kg/m^2^ for the diagnosis of hypertension in both sex.^37^ The increasing risk of having elevated/stage 1 hypertension was also found to be associated with increasing WC in young men. The optimal cut‐off values using WC was 93.50 cm in our study.

Accepted WHR value associated with the risk of hypertension by WHO has estimated 1.0 in men and 0.85 in women. In addition, in a study by Gupta and Kapoor, the WHR value was 0.90 for Indian men and 0.78 for Indian women.^33^ The WHR value for males and females were estimated to be 0.92 and 0.91, respectively in Iraq.^16^ In our study, in 35–45 age level, the estimated optimal cut‐off for WHR (0.90 for males and for 0.88 for females) to predict undiagnosed hypertension and (0.93 for males and 0.87 for females) to diagnose elevated/stage 1 hypertension stage was almost different to those studies. This deviation may be due to the considering age level.

This study has several limitations. Due to the cross‐sectional design, it is difficult to determine causality. Another limitation of this study was that neck circumference, which is an important obesity indicator, was not recorded in Shahedieh study. Despite these limitations, our study has powerful statistical results because Shahedieh reflects a large sample population‐based study in center of Iran. Since the effectiveness of obesity related markers and determining their optimal cut‐off points to predict hypertension depends on race and ethnicity, it is necessary to be assessed locally.

In conclusion, our findings showed that in Iranian adults, BMI is the most important indicator for association with undiagnosed hypertension in 35–45 years old adults. These indicators have a vital public health implication for developing countries.^38^ Hypertension is the most important risk factors for cardiovascular disease. This finding offer the prospect of an extremely effective, simple, inexpensive, and noninvasive means for a first‐level screening for hypertension. Current study also has another important finding. The best indicator to predict elevated/stage 1 hypertension stages were WC for men and BMI for women in young participants. Some differences in the present study may be due to the definition of hypertensive levels and age‐sex grouping.

## AUTHOR CONTRIBUTIONS

**Sara Jambarsang**: Conceptualization; methodology; formal analysis; writing—original draft; writing—review and editing; visualization; supervision. **Moslem Taheri Soodejani**: Writing—original draft; writing—review and editing; methodology. **Robert Tate**: Writing—original draft; writing—review and editing; methodology. **Reyhane Sefidkar**: Writing—review and editing; writing—original draft; supervision; methodology; conceptualization.

## CONFLICT OF INTEREST STATEMENT

The authors declare no conflict of interest.

## ETHICS STATEMENT

Ethics approval was obtained from the Ethics Committee of Shahid Sadoughi University of Medical Sciences (IR.SSU.SPH.REC.1401.089). The Shahedieh cohort study was conducted according to the guidelines laid down in the Declaration of Helsinki and informed consent was obtained from participants or legally authorized representatives of illiterate participants.

## TRANSPARENCY STATEMENT

The lead author Reyhane Sefidkar affirms that this manuscript is an honest, accurate, and transparent account of the study being reported; that no important aspects of the study have been omitted; and that any discrepancies from the study as planned (and, if relevant, registered) have been explained.

## Data Availability

The data that support the findings of this study are available from the corresponding author upon reasonable request.
